# Tris[4-chloro-2-(2-furylmethyl­imino­meth­yl)phenolato-κ^2^ *O* ^1^,*N*]iron(III)

**DOI:** 10.1107/S1600536808014165

**Published:** 2008-05-17

**Authors:** Ming-Qian Feng, Jing Li, Wen-Jie Wei, Chang-Hong Liu

**Affiliations:** a State Key Laboratory of Pharmaceutical Biotechnology, Nanjing University, Nanjing 210093, People’s Republic of China

## Abstract

The title complex, [Fe(C_12_H_9_ClNO_2_)_3_], is a mononuclear Schiff base iron(III) compound. The Fe atom is six-coordinated by three phenolic O and three imine N atoms from three Schiff base ligands in an octa­hedral geometry.

## Related literature

For related structures see Chiari *et al.* (1983 ▶); Hernandez-Molina *et al.* (1998 ▶); Li *et al.* (2006 ▶); Liu *et al.* (2004 ▶); Yang *et al.* (2001 ▶); You *et al.* (2005 ▶); Zhang *et al.* (2005 ▶).
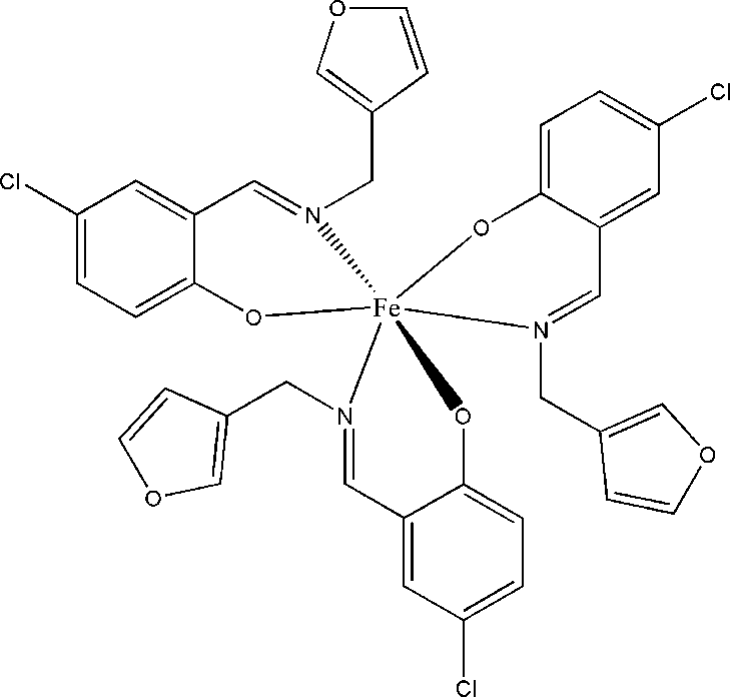


## Experimental

### 

#### Crystal data

[Fe(C_12_H_9_ClNO_2_)_3_]
*M* *_r_* = 759.81
Triclinic, 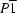

*a* = 9.622 (2) Å
*b* = 11.542 (2) Å
*c* = 16.605 (3) Å
α = 103.00 (3)°
β = 102.81 (3)°
γ = 105.50 (3)°
*V* = 1651.7 (6) Å^3^
*Z* = 2
Mo *K*α radiation
μ = 0.75 mm^−1^
*T* = 293 (2) K
0.30 × 0.20 × 0.20 mm

#### Data collection

Bruker SMART 1000 CCD area-detector diffractometer
Absorption correction: multi-scan (*SADABS*; Bruker, 2000 ▶) *T*_min_ = 0.806, *T*_max_ = 0.864
6546 measured reflections
6150 independent reflections
4172 reflections with *I* > 2σ(*I*)
*R*_int_ = 0.030

#### Refinement

*R*[*F* ^2^ > 2σ(*F* ^2^)] = 0.052
*wR*(*F* ^2^) = 0.114
*S* = 1.05
6150 reflections
442 parameters
H-atom parameters constrained
Δρ_max_ = 0.30 e Å^−3^
Δρ_min_ = −0.29 e Å^−3^

### 

Data collection: *SMART* (Bruker, 2000 ▶); cell refinement: *SAINT* (Bruker, 2000 ▶); data reduction: *SAINT*; program(s) used to solve structure: *SHELXTL* (Sheldrick, 2008 ▶); program(s) used to refine structure: *SHELXTL*; molecular graphics: *SHELXTL*; software used to prepare material for publication: *SHELXTL*.

## Supplementary Material

Crystal structure: contains datablocks global, I. DOI: 10.1107/S1600536808014165/sj2500sup1.cif

Structure factors: contains datablocks I. DOI: 10.1107/S1600536808014165/sj2500Isup2.hkl

Additional supplementary materials:  crystallographic information; 3D view; checkCIF report

## Figures and Tables

**Fig. 1.**
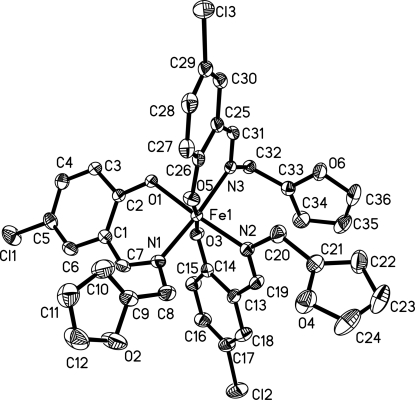
The structure of the complex with displacement parameters drawn at the 30% probability level. Hydrogen atoms have been omitted for clarity.

## supplementary crystallographic information

### Comment

Schiff base iron(III) complexes have been widely investigated due to their versatile structures and properties (Li *et al.*, 2006; Liu *et al.*, 2004; Yang *et al.*, 2001; Chiari *et al.*, 1983). We report herein the crystal structure of the title complex (I), Fig 1.

The title complex is a mononuclear Schiff base iron(III) compound. The Fe atom is six-coordinated by three phenolic O and three imine N atoms from three Schiff base ligands, in an octahedral geometry. All the coordinate bond values (Table 1) are typical and comparable with those observed in other similar Schiff base iron(III) complexes (You *et al.*, 2005; Hernandez-Molina *et al.*, 1998; Zhang *et al.*, 2005).

### Experimental

5-Chlorosalicylaldehyde (0.3 mmol, 46.8 mg), furan-2-ylmethylamine (0.3 mmol, 29.1 mg) and FeCl_3_ (0.1 mmol, 16.2 mg) were dissolved in a methanol solution (30 ml). The mixture was stirred at room temperature for 30 min to give a deep brown solution. After keeping the solution in air for a few days, deep brown crystals were formed.

### Refinement

H atoms were positioned geometrically and refined using a riding model with d(C—H) = 0.93–0.97 Å, *U*_iso_ = 1.2*U*_eq_(C) for aromatic, 0.97 Å, *U*_iso_ = 1.2*U*_eq_ (C) for CH_2_.

### Crystal data

**Table:**

[Fe(C_12_H_9_Cl_1_N_1_O_2_)_3_]	*Z* = 2
*M**_r_* = 759.81	*F*_000_ = 778
Triclinic, *P*1	*D*_x_ = 1.528 Mg m^−^^3^
*a* = 9.622 (2) Å	Mo *K*α radiation λ = 0.71073 Å
*b* = 11.542 (2) Å	Cell parameters from 2732 reflections
*c* = 16.605 (3) Å	θ = 2.3–25.1º
α = 103.00 (3)º	µ = 0.75 mm^−^^1^
β = 102.81 (3)º	*T* = 293 (2) K
γ = 105.50 (3)º	Block, brown
*V* = 1651.7 (6) Å^3^	0.30 × 0.20 × 0.20 mm

### Data collection

**Table:**

Bruker SMART 1000 CCD area-detector diffractometer	6150 independent reflections
Radiation source: fine-focus sealed tube	4172 reflections with *I* > 2σ(*I*)
Monochromator: graphite	*R*_int_ = 0.030
*T* = 293(2) K	θ_max_ = 25.5º
ω scans	θ_min_ = 1.3º
Absorption correction: multi-scan(SADABS; Bruker, 2000)	*h* = −11→0
*T*_min_ = 0.806, *T*_max_ = 0.864	*k* = −13→13
6546 measured reflections	*l* = −19→20

### Refinement

**Table:**

Refinement on *F*^2^	Secondary atom site location: difference Fourier map
Least-squares matrix: full	Hydrogen site location: inferred from neighbouring sites
*R*[*F*^2^ > 2σ(*F*^2^)] = 0.052	H-atom parameters constrained
*wR*(*F*^2^) = 0.114	*w* = 1/[σ^2^(*F*_o_^2^) + (0.0309*P*)^2^ + 1.247*P*] where *P* = (*F*_o_^2^ + 2*F*_c_^2^)/3
*S* = 1.05	(Δ/σ)_max_ < 0.001
6150 reflections	Δρ_max_ = 0.30 e Å^−^^3^
442 parameters	Δρ_min_ = −0.29 e Å^−^^3^
Primary atom site location: structure-invariant direct methods	Extinction correction: none

### Special details

**Table:**

Geometry. All e.s.d.'s (except the e.s.d. in the dihedral angle between two l.s. planes) are estimated using the full covariance matrix. The cell e.s.d.'s are taken into account individually in the estimation of e.s.d.'s in distances, angles and torsion angles; correlations between e.s.d.'s in cell parameters are only used when they are defined by crystal symmetry. An approximate (isotropic) treatment of cell e.s.d.'s is used for estimating e.s.d.'s involving l.s. planes.
Refinement. Refinement of *F*^2^ against ALL reflections. The weighted *R*-factor *wR* and goodness of fit *S* are based on *F*^2^, conventional *R*-factors *R* are based on *F*, with *F* set to zero for negative *F*^2^. The threshold expression of *F*^2^ > σ(*F*^2^) is used only for calculating *R*-factors(gt) *etc*. and is not relevant to the choice of reflections for refinement. *R*-factors based on *F*^2^ are statistically about twice as large as those based on *F*, and *R*- factors based on ALL data will be even larger.

### Fractional atomic coordinates and isotropic or equivalent isotropic displacement parameters (Å^2^)

**Table:**

	*x*	*y*	*z*	*U*_iso_*/*U*_eq_	
C1	0.8963 (4)	0.8886 (4)	0.7319 (3)	0.0377 (10)	
C2	0.8341 (4)	0.7860 (4)	0.6545 (2)	0.0332 (9)	
C3	0.8877 (4)	0.8012 (4)	0.5837 (3)	0.0408 (10)	
H3	0.8527	0.7339	0.5331	0.049*	
C4	0.9901 (5)	0.9125 (4)	0.5874 (3)	0.0451 (11)	
H4	1.0231	0.9201	0.5397	0.054*	
C5	1.0444 (5)	1.0139 (4)	0.6628 (3)	0.0461 (11)	
C6	1.0023 (5)	1.0026 (4)	0.7347 (3)	0.0450 (11)	
H6	1.0432	1.0697	0.7855	0.054*	
C7	0.8729 (5)	0.8727 (4)	0.8115 (3)	0.0407 (10)	
H7	0.9376	0.9340	0.8624	0.049*	
C8	0.7906 (5)	0.7713 (4)	0.9083 (3)	0.0477 (11)	
H8A	0.7202	0.6916	0.9054	0.057*	
H8B	0.7656	0.8383	0.9424	0.057*	
C9	0.9470 (5)	0.7789 (4)	0.9530 (3)	0.0497 (12)	
C10	1.0404 (6)	0.7212 (5)	0.9308 (3)	0.0724 (16)	
H10	1.0212	0.6598	0.8789	0.087*	
C11	1.1762 (6)	0.7712 (5)	1.0016 (4)	0.0723 (16)	
H11	1.2629	0.7490	1.0048	0.087*	
C12	1.1551 (7)	0.8537 (6)	1.0606 (4)	0.090 (2)	
H12	1.2258	0.9007	1.1141	0.108*	
C13	0.4529 (5)	0.8389 (4)	0.8092 (3)	0.0394 (10)	
C14	0.5093 (4)	0.8639 (4)	0.7410 (3)	0.0345 (9)	
C15	0.5156 (5)	0.9808 (4)	0.7261 (3)	0.0452 (11)	
H15	0.5475	0.9985	0.6799	0.054*	
C16	0.4755 (5)	1.0691 (4)	0.7783 (3)	0.0524 (12)	
H16	0.4818	1.1459	0.7677	0.063*	
C17	0.4261 (5)	1.0431 (4)	0.8460 (3)	0.0512 (12)	
C18	0.4107 (5)	0.9310 (4)	0.8610 (3)	0.0494 (12)	
H18	0.3724	0.9140	0.9054	0.059*	
C19	0.4201 (5)	0.7158 (4)	0.8207 (3)	0.0415 (10)	
H19	0.3576	0.6970	0.8548	0.050*	
C20	0.4115 (5)	0.5014 (4)	0.7962 (3)	0.0482 (11)	
H20A	0.3505	0.4444	0.7392	0.058*	
H20B	0.4970	0.4740	0.8146	0.058*	
C21	0.3201 (5)	0.4868 (4)	0.8563 (3)	0.0472 (11)	
C22	0.1753 (6)	0.4256 (5)	0.8454 (4)	0.0735 (16)	
H22	0.1004	0.3835	0.7926	0.088*	
C23	0.1561 (8)	0.4358 (6)	0.9274 (4)	0.086 (2)	
H23	0.0667	0.4029	0.9397	0.103*	
C24	0.2883 (8)	0.5005 (7)	0.9830 (4)	0.087 (2)	
H24	0.3085	0.5192	1.0427	0.104*	
C25	0.5834 (4)	0.3675 (3)	0.6264 (3)	0.0347 (9)	
C26	0.6809 (4)	0.4270 (4)	0.7118 (3)	0.0345 (9)	
C27	0.7674 (4)	0.3602 (4)	0.7489 (3)	0.0428 (10)	
H27	0.8247	0.3936	0.8070	0.051*	
C28	0.7684 (5)	0.2470 (4)	0.7012 (3)	0.0454 (11)	
H28	0.8271	0.2051	0.7267	0.054*	
C29	0.6820 (5)	0.1950 (4)	0.6148 (3)	0.0436 (11)	
C30	0.5872 (4)	0.2511 (4)	0.5786 (3)	0.0387 (10)	
H30	0.5246	0.2125	0.5218	0.046*	
C31	0.4788 (4)	0.4211 (4)	0.5857 (3)	0.0341 (9)	
H31	0.4130	0.3721	0.5311	0.041*	
C32	0.3452 (4)	0.5605 (4)	0.5631 (2)	0.0339 (9)	
H32A	0.3854	0.6465	0.5621	0.041*	
H32B	0.3128	0.5054	0.5040	0.041*	
C33	0.2134 (4)	0.5477 (4)	0.5959 (3)	0.0372 (10)	
C34	0.1434 (5)	0.6272 (5)	0.6248 (3)	0.0555 (13)	
H34	0.1725	0.7133	0.6319	0.067*	
C35	0.0168 (6)	0.5541 (6)	0.6421 (3)	0.0671 (15)	
H35	−0.0525	0.5834	0.6640	0.080*	
C36	0.0153 (6)	0.4367 (6)	0.6216 (3)	0.0676 (15)	
H36	−0.0584	0.3689	0.6254	0.081*	
Cl1	1.16738 (17)	1.15609 (12)	0.66343 (9)	0.0758 (4)	
Cl2	0.38023 (18)	1.15945 (13)	0.91252 (10)	0.0792 (5)	
Cl3	0.69996 (15)	0.05941 (11)	0.55247 (9)	0.0668 (4)	
Fe1	0.61221 (6)	0.65594 (5)	0.72051 (3)	0.02604 (14)	
N1	0.7708 (4)	0.7817 (3)	0.8190 (2)	0.0370 (8)	
N2	0.4705 (4)	0.6291 (3)	0.7876 (2)	0.0351 (8)	
N3	0.4665 (3)	0.5287 (3)	0.61637 (19)	0.0295 (7)	
O1	0.7387 (3)	0.6767 (2)	0.64791 (16)	0.0370 (7)	
O2	1.0136 (4)	0.8618 (3)	1.0329 (2)	0.0808 (12)	
O3	0.5480 (3)	0.7841 (2)	0.68880 (17)	0.0358 (6)	
O4	0.3940 (4)	0.5377 (4)	0.9423 (2)	0.0744 (11)	
O5	0.6944 (3)	0.5392 (2)	0.75910 (17)	0.0394 (7)	
O6	0.1379 (3)	0.4282 (3)	0.5938 (2)	0.0537 (8)	

### Atomic displacement parameters (Å^2^)

**Table:**

	*U*^11^	*U*^22^	*U*^33^	*U*^12^	*U*^13^	*U*^23^
C1	0.036 (2)	0.034 (2)	0.038 (2)	0.0084 (19)	0.0110 (19)	0.0072 (19)
C2	0.031 (2)	0.032 (2)	0.036 (2)	0.0112 (18)	0.0114 (18)	0.0072 (18)
C3	0.038 (2)	0.040 (2)	0.040 (3)	0.009 (2)	0.017 (2)	0.004 (2)
C4	0.044 (3)	0.045 (3)	0.048 (3)	0.011 (2)	0.019 (2)	0.018 (2)
C5	0.043 (3)	0.036 (2)	0.053 (3)	0.003 (2)	0.011 (2)	0.018 (2)
C6	0.047 (3)	0.037 (2)	0.039 (3)	0.006 (2)	0.008 (2)	0.002 (2)
C7	0.044 (3)	0.034 (2)	0.032 (2)	0.008 (2)	0.006 (2)	−0.0016 (18)
C8	0.050 (3)	0.055 (3)	0.035 (2)	0.017 (2)	0.012 (2)	0.009 (2)
C9	0.051 (3)	0.053 (3)	0.036 (3)	0.014 (2)	0.007 (2)	0.006 (2)
C10	0.075 (4)	0.087 (4)	0.056 (3)	0.037 (3)	0.020 (3)	0.008 (3)
C11	0.061 (4)	0.085 (4)	0.081 (4)	0.040 (3)	0.017 (3)	0.028 (3)
C12	0.063 (4)	0.100 (5)	0.075 (4)	0.037 (4)	−0.016 (3)	−0.009 (4)
C13	0.039 (2)	0.039 (2)	0.045 (3)	0.0142 (19)	0.020 (2)	0.012 (2)
C14	0.030 (2)	0.034 (2)	0.037 (2)	0.0075 (18)	0.0092 (18)	0.0103 (19)
C15	0.057 (3)	0.037 (2)	0.049 (3)	0.018 (2)	0.023 (2)	0.017 (2)
C16	0.063 (3)	0.037 (3)	0.059 (3)	0.022 (2)	0.021 (3)	0.009 (2)
C17	0.055 (3)	0.045 (3)	0.054 (3)	0.025 (2)	0.020 (2)	0.004 (2)
C18	0.055 (3)	0.055 (3)	0.048 (3)	0.029 (2)	0.025 (2)	0.012 (2)
C19	0.044 (3)	0.049 (3)	0.037 (2)	0.016 (2)	0.021 (2)	0.014 (2)
C20	0.054 (3)	0.044 (3)	0.054 (3)	0.014 (2)	0.025 (2)	0.022 (2)
C21	0.053 (3)	0.052 (3)	0.045 (3)	0.021 (2)	0.018 (2)	0.023 (2)
C22	0.058 (4)	0.085 (4)	0.074 (4)	0.006 (3)	0.026 (3)	0.031 (3)
C23	0.078 (4)	0.119 (6)	0.104 (5)	0.043 (4)	0.058 (4)	0.074 (5)
C24	0.107 (5)	0.141 (6)	0.074 (4)	0.079 (5)	0.058 (4)	0.075 (4)
C25	0.031 (2)	0.029 (2)	0.042 (2)	0.0057 (17)	0.0120 (19)	0.0102 (18)
C26	0.027 (2)	0.032 (2)	0.046 (3)	0.0076 (18)	0.0144 (19)	0.0141 (19)
C27	0.035 (2)	0.045 (3)	0.049 (3)	0.012 (2)	0.011 (2)	0.020 (2)
C28	0.036 (2)	0.043 (3)	0.067 (3)	0.021 (2)	0.017 (2)	0.026 (2)
C29	0.038 (2)	0.031 (2)	0.063 (3)	0.0100 (19)	0.023 (2)	0.010 (2)
C30	0.035 (2)	0.031 (2)	0.047 (3)	0.0074 (19)	0.014 (2)	0.010 (2)
C31	0.030 (2)	0.032 (2)	0.032 (2)	0.0019 (17)	0.0099 (18)	0.0056 (18)
C32	0.033 (2)	0.038 (2)	0.032 (2)	0.0120 (18)	0.0106 (18)	0.0110 (18)
C33	0.035 (2)	0.041 (2)	0.034 (2)	0.0121 (19)	0.0076 (19)	0.0122 (19)
C34	0.049 (3)	0.053 (3)	0.070 (3)	0.029 (2)	0.017 (3)	0.015 (3)
C35	0.053 (3)	0.094 (4)	0.073 (4)	0.040 (3)	0.033 (3)	0.027 (3)
C36	0.045 (3)	0.093 (4)	0.077 (4)	0.019 (3)	0.034 (3)	0.037 (3)
Cl1	0.0852 (10)	0.0462 (7)	0.0765 (10)	−0.0099 (7)	0.0222 (8)	0.0222 (7)
Cl2	0.0968 (11)	0.0659 (9)	0.0840 (10)	0.0482 (8)	0.0408 (9)	0.0015 (7)
Cl3	0.0663 (8)	0.0420 (7)	0.0972 (11)	0.0285 (6)	0.0331 (8)	0.0098 (7)
Fe1	0.0258 (3)	0.0256 (3)	0.0263 (3)	0.0081 (2)	0.0099 (2)	0.0055 (2)
N1	0.041 (2)	0.0364 (19)	0.0294 (19)	0.0086 (16)	0.0126 (16)	0.0050 (15)
N2	0.0360 (19)	0.0362 (19)	0.0355 (19)	0.0126 (16)	0.0135 (16)	0.0119 (16)
N3	0.0255 (17)	0.0315 (18)	0.0330 (18)	0.0096 (14)	0.0117 (14)	0.0094 (15)
O1	0.0350 (15)	0.0317 (15)	0.0401 (16)	0.0045 (12)	0.0196 (13)	0.0024 (13)
O2	0.075 (3)	0.084 (3)	0.055 (2)	0.039 (2)	−0.012 (2)	−0.0175 (19)
O3	0.0424 (16)	0.0338 (15)	0.0377 (16)	0.0161 (13)	0.0203 (13)	0.0109 (13)
O4	0.066 (2)	0.115 (3)	0.053 (2)	0.030 (2)	0.0212 (19)	0.043 (2)
O5	0.0408 (16)	0.0350 (16)	0.0375 (16)	0.0144 (13)	0.0053 (13)	0.0059 (13)
O6	0.0452 (18)	0.0497 (19)	0.073 (2)	0.0140 (15)	0.0318 (17)	0.0198 (17)

### Geometric parameters (Å, °)

**Table:**

C1—C2	1.416 (5)	C20—H20A	0.9700
C1—C6	1.417 (5)	C20—H20B	0.9700
C1—C7	1.433 (5)	C21—C22	1.333 (6)
C2—O1	1.312 (4)	C21—O4	1.356 (5)
C2—C3	1.412 (5)	C22—C23	1.398 (7)
C3—C4	1.373 (5)	C22—H22	0.9300
C3—H3	0.9300	C23—C24	1.299 (8)
C4—C5	1.390 (6)	C23—H23	0.9300
C4—H4	0.9300	C24—O4	1.371 (6)
C5—C6	1.364 (6)	C24—H24	0.9300
C5—Cl1	1.744 (4)	C25—C26	1.411 (5)
C6—H6	0.9300	C25—C30	1.412 (5)
C7—N1	1.283 (5)	C25—C31	1.440 (5)
C7—H7	0.9300	C26—O5	1.311 (4)
C8—N1	1.488 (5)	C26—C27	1.412 (5)
C8—C9	1.493 (6)	C27—C28	1.371 (6)
C8—H8A	0.9700	C27—H27	0.9300
C8—H8B	0.9700	C28—C29	1.387 (6)
C9—C10	1.320 (6)	C28—H28	0.9300
C9—O2	1.345 (5)	C29—C30	1.365 (6)
C10—C11	1.426 (7)	C29—Cl3	1.746 (4)
C10—H10	0.9300	C30—H30	0.9300
C11—C12	1.296 (7)	C31—N3	1.280 (5)
C11—H11	0.9300	C31—H31	0.9300
C12—O2	1.373 (6)	C32—C33	1.473 (5)
C12—H12	0.9300	C32—N3	1.482 (5)
C13—C14	1.413 (5)	C32—H32A	0.9700
C13—C18	1.417 (5)	C32—H32B	0.9700
C13—C19	1.437 (5)	C33—C34	1.342 (6)
C14—O3	1.306 (4)	C33—O6	1.366 (5)
C14—C15	1.413 (5)	C34—C35	1.412 (7)
C15—C16	1.378 (6)	C34—H34	0.9300
C15—H15	0.9300	C35—C36	1.315 (7)
C16—C17	1.376 (6)	C35—H35	0.9300
C16—H16	0.9300	C36—O6	1.377 (5)
C17—C18	1.349 (6)	C36—H36	0.9300
C17—Cl2	1.756 (4)	Fe1—O3	1.882 (3)
C18—H18	0.9300	Fe1—O5	1.894 (3)
C19—N2	1.291 (5)	Fe1—O1	1.902 (3)
C19—H19	0.9300	Fe1—N1	1.937 (3)
C20—C21	1.477 (6)	Fe1—N3	1.948 (3)
C20—N2	1.482 (5)	Fe1—N2	1.950 (3)
			
C2—C1—C6	120.1 (4)	C22—C23—H23	126.9
C2—C1—C7	120.7 (4)	C23—C24—O4	111.3 (5)
C6—C1—C7	118.5 (4)	C23—C24—H24	124.3
O1—C2—C3	119.2 (3)	O4—C24—H24	124.3
O1—C2—C1	123.4 (3)	C26—C25—C30	119.5 (4)
C3—C2—C1	117.2 (4)	C26—C25—C31	122.3 (3)
C4—C3—C2	121.9 (4)	C30—C25—C31	118.2 (4)
C4—C3—H3	119.0	O5—C26—C25	124.1 (4)
C2—C3—H3	119.0	O5—C26—C27	118.1 (4)
C3—C4—C5	119.8 (4)	C25—C26—C27	117.7 (4)
C3—C4—H4	120.1	C28—C27—C26	121.3 (4)
C5—C4—H4	120.1	C28—C27—H27	119.3
C6—C5—C4	120.8 (4)	C26—C27—H27	119.3
C6—C5—Cl1	120.7 (3)	C27—C28—C29	120.1 (4)
C4—C5—Cl1	118.5 (3)	C27—C28—H28	120.0
C5—C6—C1	120.1 (4)	C29—C28—H28	120.0
C5—C6—H6	120.0	C30—C29—C28	120.4 (4)
C1—C6—H6	120.0	C30—C29—Cl3	120.4 (4)
N1—C7—C1	126.0 (4)	C28—C29—Cl3	119.2 (3)
N1—C7—H7	117.0	C29—C30—C25	120.6 (4)
C1—C7—H7	117.0	C29—C30—H30	119.7
N1—C8—C9	113.5 (4)	C25—C30—H30	119.7
N1—C8—H8A	108.9	N3—C31—C25	127.1 (4)
C9—C8—H8A	108.9	N3—C31—H31	116.5
N1—C8—H8B	108.9	C25—C31—H31	116.5
C9—C8—H8B	108.9	C33—C32—N3	112.6 (3)
H8A—C8—H8B	107.7	C33—C32—H32A	109.1
C10—C9—O2	109.8 (4)	N3—C32—H32A	109.1
C10—C9—C8	133.6 (4)	C33—C32—H32B	109.1
O2—C9—C8	116.6 (4)	N3—C32—H32B	109.1
C9—C10—C11	106.9 (5)	H32A—C32—H32B	107.8
C9—C10—H10	126.5	C34—C33—O6	110.3 (4)
C11—C10—H10	126.5	C34—C33—C32	134.5 (4)
C12—C11—C10	106.5 (5)	O6—C33—C32	115.2 (3)
C12—C11—H11	126.8	C33—C34—C35	106.4 (4)
C10—C11—H11	126.8	C33—C34—H34	126.8
C11—C12—O2	110.4 (5)	C35—C34—H34	126.8
C11—C12—H12	124.8	C36—C35—C34	107.3 (4)
O2—C12—H12	124.8	C36—C35—H35	126.4
C14—C13—C18	120.0 (4)	C34—C35—H35	126.4
C14—C13—C19	120.9 (4)	C35—C36—O6	110.7 (5)
C18—C13—C19	118.7 (4)	C35—C36—H36	124.7
O3—C14—C13	124.4 (4)	O6—C36—H36	124.7
O3—C14—C15	118.5 (4)	O3—Fe1—O5	174.53 (12)
C13—C14—C15	117.0 (4)	O3—Fe1—O1	86.76 (11)
C16—C15—C14	121.6 (4)	O5—Fe1—O1	91.70 (12)
C16—C15—H15	119.2	O3—Fe1—N1	89.84 (13)
C14—C15—H15	119.2	O5—Fe1—N1	84.94 (13)
C17—C16—C15	119.7 (4)	O1—Fe1—N1	91.02 (13)
C17—C16—H16	120.1	O3—Fe1—N3	91.31 (12)
C15—C16—H16	120.1	O5—Fe1—N3	93.76 (12)
C18—C17—C16	121.5 (4)	O1—Fe1—N3	84.11 (12)
C18—C17—Cl2	120.2 (4)	N1—Fe1—N3	174.93 (13)
C16—C17—Cl2	118.3 (4)	O3—Fe1—N2	92.00 (13)
C17—C18—C13	120.1 (4)	O5—Fe1—N2	89.87 (13)
C17—C18—H18	120.0	O1—Fe1—N2	175.99 (13)
C13—C18—H18	120.0	N1—Fe1—N2	92.79 (14)
N2—C19—C13	125.3 (4)	N3—Fe1—N2	92.11 (13)
N2—C19—H19	117.3	C7—N1—C8	115.3 (3)
C13—C19—H19	117.3	C7—N1—Fe1	122.9 (3)
C21—C20—N2	117.4 (4)	C8—N1—Fe1	121.5 (3)
C21—C20—H20A	107.9	C19—N2—C20	118.9 (3)
N2—C20—H20A	107.9	C19—N2—Fe1	122.7 (3)
C21—C20—H20B	107.9	C20—N2—Fe1	118.3 (3)
N2—C20—H20B	107.9	C31—N3—C32	116.8 (3)
H20A—C20—H20B	107.2	C31—N3—Fe1	123.0 (3)
C22—C21—O4	109.0 (4)	C32—N3—Fe1	119.8 (2)
C22—C21—C20	133.7 (5)	C2—O1—Fe1	123.3 (2)
O4—C21—C20	117.1 (4)	C9—O2—C12	106.5 (4)
C21—C22—C23	107.9 (5)	C14—O3—Fe1	122.8 (2)
C21—C22—H22	126.1	C21—O4—C24	105.5 (4)
C23—C22—H22	126.1	C26—O5—Fe1	126.4 (3)
C24—C23—C22	106.2 (5)	C33—O6—C36	105.4 (4)
C24—C23—H23	126.9		
			
C6—C1—C2—O1	178.1 (4)	O5—Fe1—N1—C7	119.8 (3)
C7—C1—C2—O1	8.2 (6)	O1—Fe1—N1—C7	28.2 (3)
C6—C1—C2—C3	3.0 (6)	N3—Fe1—N1—C7	44.5 (17)
C7—C1—C2—C3	−166.9 (4)	N2—Fe1—N1—C7	−150.6 (3)
O1—C2—C3—C4	−178.6 (4)	O3—Fe1—N1—C8	127.6 (3)
C1—C2—C3—C4	−3.3 (6)	O5—Fe1—N1—C8	−54.0 (3)
C2—C3—C4—C5	0.4 (7)	O1—Fe1—N1—C8	−145.6 (3)
C3—C4—C5—C6	2.9 (7)	N3—Fe1—N1—C8	−129.3 (15)
C3—C4—C5—Cl1	−177.0 (3)	N2—Fe1—N1—C8	35.6 (3)
C4—C5—C6—C1	−3.2 (7)	C13—C19—N2—C20	−172.4 (4)
Cl1—C5—C6—C1	176.8 (3)	C13—C19—N2—Fe1	3.8 (6)
C2—C1—C6—C5	0.1 (6)	C21—C20—N2—C19	−11.3 (6)
C7—C1—C6—C5	170.3 (4)	C21—C20—N2—Fe1	172.3 (3)
C2—C1—C7—N1	−17.6 (7)	O3—Fe1—N2—C19	−25.3 (3)
C6—C1—C7—N1	172.3 (4)	O5—Fe1—N2—C19	149.5 (3)
N1—C8—C9—C10	−49.9 (8)	O1—Fe1—N2—C19	−97.2 (18)
N1—C8—C9—O2	129.2 (4)	N1—Fe1—N2—C19	64.6 (3)
O2—C9—C10—C11	−0.1 (6)	N3—Fe1—N2—C19	−116.7 (3)
C8—C9—C10—C11	179.0 (5)	O3—Fe1—N2—C20	150.9 (3)
C9—C10—C11—C12	0.3 (7)	O5—Fe1—N2—C20	−34.2 (3)
C10—C11—C12—O2	−0.3 (8)	O1—Fe1—N2—C20	79.0 (19)
C18—C13—C14—O3	−178.5 (4)	N1—Fe1—N2—C20	−119.2 (3)
C19—C13—C14—O3	−6.1 (6)	N3—Fe1—N2—C20	59.5 (3)
C18—C13—C14—C15	−2.2 (6)	C25—C31—N3—C32	−177.7 (3)
C19—C13—C14—C15	170.2 (4)	C25—C31—N3—Fe1	9.8 (5)
O3—C14—C15—C16	179.6 (4)	C33—C32—N3—C31	103.3 (4)
C13—C14—C15—C16	3.1 (6)	C33—C32—N3—Fe1	−83.9 (4)
C14—C15—C16—C17	−0.9 (7)	O3—Fe1—N3—C31	160.0 (3)
C15—C16—C17—C18	−2.3 (7)	O5—Fe1—N3—C31	−18.0 (3)
C15—C16—C17—Cl2	178.6 (4)	O1—Fe1—N3—C31	73.4 (3)
C16—C17—C18—C13	3.2 (7)	N1—Fe1—N3—C31	57.0 (17)
Cl2—C17—C18—C13	−177.7 (3)	N2—Fe1—N3—C31	−108.0 (3)
C14—C13—C18—C17	−0.8 (7)	O3—Fe1—N3—C32	−12.4 (3)
C19—C13—C18—C17	−173.4 (4)	O5—Fe1—N3—C32	169.7 (3)
C14—C13—C19—N2	18.0 (7)	O1—Fe1—N3—C32	−99.0 (3)
C18—C13—C19—N2	−169.5 (4)	N1—Fe1—N3—C32	−115.3 (16)
N2—C20—C21—C22	113.5 (6)	N2—Fe1—N3—C32	79.7 (3)
N2—C20—C21—O4	−71.4 (5)	C3—C2—O1—Fe1	−160.3 (3)
O4—C21—C22—C23	−1.0 (6)	C1—C2—O1—Fe1	24.7 (5)
C20—C21—C22—C23	174.4 (5)	O3—Fe1—O1—C2	52.8 (3)
C21—C22—C23—C24	−0.6 (7)	O5—Fe1—O1—C2	−122.0 (3)
C22—C23—C24—O4	2.0 (8)	N1—Fe1—O1—C2	−37.0 (3)
C30—C25—C26—O5	174.6 (3)	N3—Fe1—O1—C2	144.4 (3)
C31—C25—C26—O5	−5.6 (6)	N2—Fe1—O1—C2	124.9 (18)
C30—C25—C26—C27	−6.6 (5)	C10—C9—O2—C12	−0.1 (6)
C31—C25—C26—C27	173.3 (3)	C8—C9—O2—C12	−179.4 (5)
O5—C26—C27—C28	−174.7 (4)	C11—C12—O2—C9	0.3 (7)
C25—C26—C27—C28	6.4 (6)	C13—C14—O3—Fe1	−26.3 (5)
C26—C27—C28—C29	−0.9 (6)	C15—C14—O3—Fe1	157.5 (3)
C27—C28—C29—C30	−4.7 (6)	O5—Fe1—O3—C14	−73.8 (13)
C27—C28—C29—Cl3	173.5 (3)	O1—Fe1—O3—C14	−147.6 (3)
C28—C29—C30—C25	4.4 (6)	N1—Fe1—O3—C14	−56.5 (3)
Cl3—C29—C30—C25	−173.7 (3)	N3—Fe1—O3—C14	128.4 (3)
C26—C25—C30—C29	1.3 (6)	N2—Fe1—O3—C14	36.2 (3)
C31—C25—C30—C29	−178.5 (4)	C22—C21—O4—C24	2.1 (6)
C26—C25—C31—N3	5.2 (6)	C20—C21—O4—C24	−174.1 (4)
C30—C25—C31—N3	−174.9 (4)	C23—C24—O4—C21	−2.6 (7)
N3—C32—C33—C34	119.8 (5)	C25—C26—O5—Fe1	−9.7 (5)
N3—C32—C33—O6	−64.0 (4)	C27—C26—O5—Fe1	171.4 (3)
O6—C33—C34—C35	0.1 (5)	O3—Fe1—O5—C26	−139.4 (12)
C32—C33—C34—C35	176.5 (4)	O1—Fe1—O5—C26	−65.9 (3)
C33—C34—C35—C36	−1.2 (6)	N1—Fe1—O5—C26	−156.7 (3)
C34—C35—C36—O6	1.9 (6)	N3—Fe1—O5—C26	18.3 (3)
C1—C7—N1—C8	167.1 (4)	N2—Fe1—O5—C26	110.4 (3)
C1—C7—N1—Fe1	−7.1 (6)	C34—C33—O6—C36	1.0 (5)
C9—C8—N1—C7	−47.8 (5)	C32—C33—O6—C36	−176.2 (4)
C9—C8—N1—Fe1	126.5 (3)	C35—C36—O6—C33	−1.8 (6)
O3—Fe1—N1—C7	−58.6 (3)		

## References

[bb1] Bruker (2000). *SMART*, *SAINT* and *SADABS* Bruker AXS Inc., Madison, Wisconsin, USA.

[bb2] Chiari, B., Piovesana, O., Tarantelli, T. & Zanazzi, P. F. (1983). *Inorg. Chem.* **22**, 2781–2784.

[bb3] Hernandez-Molina, R., Mederos, A., Dominguez, S., Gili, P., Ruiz-Perez, C., Castineiras, A., Solans, X., Lloret, F. & Real, J. A. (1998). *Inorg. Chem.* **37**, 5102–5108.

[bb4] Li, Y.-G., Zhu, H.-L., Chen, X.-Z., Song, Y. & Huang, W.-Q. (2006). *Synth. React. Inorg. Met.-Org. Nano-Met. Chem.* **36**, 353–357.

[bb5] Liu, Z.-D., Tan, M.-Y. & Zhu, H.-L. (2004). *Acta Cryst.* E**60**, m910–m911.

[bb6] Sheldrick, G. M. (2008). *Acta Cryst.* A**64**, 112–122. 10.1107/S0108767307043930 18156677

[bb7] Yang, S.-P., Tong, Y.-X., Zhu, H.-L., Cao, H., Chen, X.-M. & Ji, L.-N. (2001). *Polyhedron*, **20**, 223–229.

[bb8] You, Z.-L., Tang, L.-L. & Zhu, H.-L. (2005). *Acta Cryst.* E**61**, m36–m38.

[bb9] Zhang, J.-H., Xi, Y., Wang, C., Li, J., Zhang, F.-X. & Ng, S. W. (2005). *Acta Cryst.* E**61**, m2338–m2339.

